# Impacts of Yacon Syrup (*Smallanthus sonchifolius*) on Human Health: A Systematic Review of Scientific Evidence from the Last Decade

**DOI:** 10.3390/nu17050888

**Published:** 2025-03-01

**Authors:** Marcos F. Pereira, Igor de Codes Soares, Marília Magalhães Cabral, Paula A. de Freitas, Gabriel M. A. Sousa, Saulo Chaves Magalhães, Antônio Augusto Ferreira Carioca, Maria Rayane C. de Oliveira, Francisco Ernani A. Magalhães, Ariclecio C. de Oliveira, Renalison Farias-Pereira, Keciany Alves de Oliveira

**Affiliations:** 1 Higher Institute of Biomedical Sciences, State University of Ceara-UECE, Fortaleza 60714-903, CE, Brazil; marquinhos.pereira@aluno.uece.br (M.F.P.); igorcodes@gmail.com (I.d.C.S.); gabrielzinho.martins@aluno.uece.br (G.M.A.S.); saulo.chaves@aluno.uece.br (S.C.M.); ariclecio.oliveira@uece.br (A.C.d.O.); 2 Postgraduate Program in Nutrition and Health, State University of Ceara-UECE, Fortaleza 60714-903, CE, Brazil; mariliamaga@gmail.com (M.M.C.); paulaalexandref@gmail.com (P.A.d.F.); rayane.correia@uece.br (M.R.C.d.O.); ernani.magalhaes@uece.br (F.E.A.M.); 3 Department of Nutrition, Postgraduate Studies in Public Health, University of Fortaleza, Fortaleza 60811-905, CE, Brazil; aafc7@hotmail.com; 4 Department of Biological Sciences, The Dorothy and George Hennings College of Science, Mathematics and Technology, Kean University, Union, NJ 07083, USA; rfariasp@kean.edu

**Keywords:** yacon syrup, fructooligosaccharides, metabolic health, gut microbiota, systematic review, insulin sensitivity, satiety, dietary supplements

## Abstract

Background/Objectives: Yacon syrup (*Smallanthus sonchifolius*) has gained attention due to its high concentration of fructooligosaccharides (FOSs) and associated health benefits. This systematic review aimed to evaluate the effects of yacon syrup on metabolic parameters and intestinal health in humans over the last decade. Methods: Following PRISMA guidelines, we conducted a systematic search in databases, including Medline (PubMed), Science Direct, Embase, Scopus, and SciELO, up to October 2024. Inclusion criteria focused on clinical trials examining the impact of yacon syrup on glycemic control, lipid profile, insulin sensitivity, appetite regulation, and gut microbiota in healthy, overweight, or obese individuals. Seven studies met the inclusion criteria, encompassing 161 participants from diverse populations. Results: Yacon syrup supplementation demonstrated significant reductions in fasting insulin, HOMA-IR, and LDL cholesterol, alongside improvements in satiety and intestinal transit time. Acute supplementation with yacon syrup had inconsistent results for postprandial glycemia and insulin levels, probably due to prior individual gut microbiota composition. Longer interventions with yacon syrup were associated with enhanced microbiota modulation and appetite regulation, particularly in women. Mild gastrointestinal discomfort was reported, but with the continued use of yacon syrup, the symptoms decreased. Yacon syrup presents promising health benefits, including improved insulin sensitivity, weight management, and gut health. However, further research is needed to establish optimal dosing and long-term safety. Conclusions: This review highlights the potential of yacon syrup as a functional supplement for metabolic and gastrointestinal health.

## 1. Introduction

*Smallanthus sonchifolius*, known as yacon, is an herbaceous plant from the Asteraceae family, originating from the Andean regions of South America [1], and has been used for many centuries by these people as a source of food and for medicinal practices [2] and later this practice spread to countries on different continents [3].

Yacon is used as food and nutraceutical, having many of its plant parts used as extracts, flours, or syrup. For example, yacon roots (potatoes) are nutrient-rich food with antimicrobial, antifungal, antihyperglycemic, and antioxidant properties [1]. Yacon roots are widely used in the production of various food products, such as syrups, flours, diet cookies, and yogurts due to their sweet flavor and functional and nutritional values [4,5]. Other yacon parts are also rich in nutrients with potential beneficial effects on human health: leaves have a high concentration of micronutrients (Fe, Zn, Mn, Cu, Co, and Ni) with antioxidant, cytoprotective and anti-hyperglycemic, antibacterial properties [6,7,8,9]; flowers also have antioxidant properties, probably due to its phenolic compounds content (e.g., myricetin and gallic acid) [10]. Therefore, yacon is an emerging potential crop for human nutrition and health, and its potato products can be easily included in the human diet due to their sensory properties [5].

The nutritional composition of yacon potatoes is notable for its high concentration of fructooligosaccharides (FOSs), fructans, inulin, and phenolic compounds. These bioactive compounds may promote health benefits such as improving glycemic homeostasis and intestinal health [11,12,13]. For example, FOS selectively stimulates the growth of beneficial bacteria (e.g., *bifidobacteria* and *lactobacilli*) in the colon, thereby increasing the production of short-chain fatty acids (SCFA) such as acetate, propionate, and butyrate [14,15]. These SCFA are rapidly absorbed by the intestinal mucosa and may exert systemic effects, including regulation of glucose and lipid metabolism. Moreover, they have been linked to improved insulin sensitivity and glycemic control—partly through the promotion of satiety hormones like GLP-1 and PYY, alongside modulation of inflammatory pathways [16].

There are studies suggesting the potential effects of yacon potatoes as extracts, flour, and syrup on metabolic parameters. The consumption of yacon potato extract has improved lipid profiles by reducing triglycerides and total cholesterol, increasing HDL cholesterol by inhibiting the enzyme HMG-CoA reductase, and activating fatty acid oxidation in hyperlipidemic rats [17]. Yacon potato flour also has potential beneficial effects against obesity by reducing weight gain, visceral fat, insulin resistance, and inflammation in adipose tissue in high-fat-diet-fed rats [18]. Yacon syrup powder reduced blood glucose, serum triglycerides, and colon inflammation, as seen by the reduction in interleukin 23 (IL-23) levels and leukocyte infiltration in the colon of colitis-induced mice, suggesting a promising anti-inflammatory effect of yacon syrup [19]. These nutritional values and functional properties of yacon potatoes, especially yacon syrup that can be used as a low-calorie sweetener, may help to prevent obesity and associated diseases in humans.

Although the evidence of the effects of yacon syrup in humans is still limited, there are studies on its effects on increasing satiety, accelerating colonic transit, and reducing metabolic parameters of obesity [4,20]. In obese and slightly dyslipidemic women, the consumption of yacon syrup reduced body weight, waist circumference, body mass index (BMI), LDL cholesterol, and fasting serum insulin levels with an increased defecation frequency and satiety [21]. Another study also suggests the potential use of yacon syrup in gastrointestinal-related diseases, since yacon syrup increased colonic transit in healthy subjects [22]. Since the last review studies on yacon as a functional food were published in 2016 and 2019 [4,20], this systematic review presents the most recent studies related to yacon syrup and its impacts on human health, including obesity-related parameters.

Given the rising prevalence of obesity, diabetes, dyslipidemia, and metabolic syndrome, along with their associated health risks, dietary modifications and the incorporation of functional foods have gained increasing attention in disease prevention and management [23]. While previous reviews have explored other forms of yacon, none have specifically focused on yacon in syrup form and its metabolic effects [24]. This systematic review aims to address this gap by synthesizing the latest scientific evidence on the influence of yacon syrup on metabolic parameters, gut health, and its potential role as an anti-obesity intervention. To the best of our knowledge, this is the first systematic review dedicated exclusively to yacon syrup and its associated health benefits.

## 2. Methods

### 2.1. Research Question, Protocol and Registration

This systematic review aims to answer the following research question: What are the beneficial effects of yacon syrup consumption in humans? This review follows the Preferred Reporting Items for Systematic Reviews and Meta-Analyses (PRISMA) guidelines [25,26]. The protocol for this systematic review was registered in the International Prospective Register of Systematic Reviews (PROSPERO) registration number CRD42024586919.

The PRISMA 2020 Checklist used in this review is available in the Supplementary Materials (Table S1).

### 2.2. Literature Search

To collect as many studies as possible on the effects of yacon syrup in humans, the following search terms were used: ‘yacon syrup’ AND (‘blood glucose’ OR insulin OR diabet OR obesity OR ‘satiety response’ OR lipids OR dyslipidemias OR ‘gut microbiota’). After defining the search terms, two authors independently carried out the search in the Medline (PubMed), Science Direct, Embase, Scopus, and SciELO databases, covering all studies published until October 2024. No additional date restrictions were applied beyond this period to ensure the inclusion of all relevant studies available.

### 2.3. Eligibility Criteria

#### 2.3.1. Inclusion Criteria

Inclusion criteria for this systematic review were established using the PICO (Population, Intervention, Comparison, and Outcome) framework to assess the effects of yacon syrup on metabolic parameters in humans. The studies included met the following criteria: (i) population: clinical studies conducted in healthy, overweight, and/or obese individuals; (ii) intervention: studies evaluating the effects of yacon syrup supplementation; (iii) outcomes: studies assessing the impact of yacon syrup on obesity, lipid profile, glycemia, insulin levels, insulin resistance, satiety response, and intestinal microbiota; (iv) study design: only peer-reviewed articles published in scientific journals; and (v) language: articles written in English to ensure the selection of high-quality, widely accessible studies.

#### 2.3.2. Exclusion Criteria

The following criteria were used to exclude articles from this systematic review: (i) review articles, book chapters, theses, letters, personal opinions, conference abstracts, and patents; (ii) studies carried out with non-human populations or that addressed sensory aspects of yacon syrup; and (iii) articles that did not include intervention programs, pre- or post-intervention evaluation, or that did not present a comparable group.

### 2.4. Study Selection and Data Extraction

The selection of studies based on the title and abstract of the articles was carried out using Rayyan (https://rayyan.ai/—accessed on 29 January 2025) [27]. Two researchers independently screened all abstracts and checked them against the inclusion/exclusion criteria. Disagreements were resolved by a third investigator. The full text of the selected articles was read to verify compliance with the eligibility criteria by two researchers independently, and similarly, disagreements were resolved by consensus.

We extracted data for (1) study characteristics (country, population, and study design), (2) intervention details (type of yacon syrup, dose, and duration), and (3) measured outcomes (anthropometric and metabolic parameters, glycemic markers, appetite-related hormones, gut microbiota changes, and adverse events). Because the included studies varied in design and endpoints, no meta-analysis was performed; instead, we conducted a narrative synthesis of the results. Two authors independently extracted study characteristics (as shown in Table 1) and outcome data using prespecified criteria for search, data extraction, and quality assessment. Discrepancies were resolved by consensus with a third author.

### 2.5. Quality Analysis

For the risk-of-bias assessment, we used RoB 2, following the guidelines of the Cochrane Handbook, since all included studies were randomized trials [28]. Due to the high heterogeneity of the results, we opted for a narrative synthesis, and a meta-analysis was not conducted.

## 3. Results

### 3.1. Selected Studies

**Figure 1:**
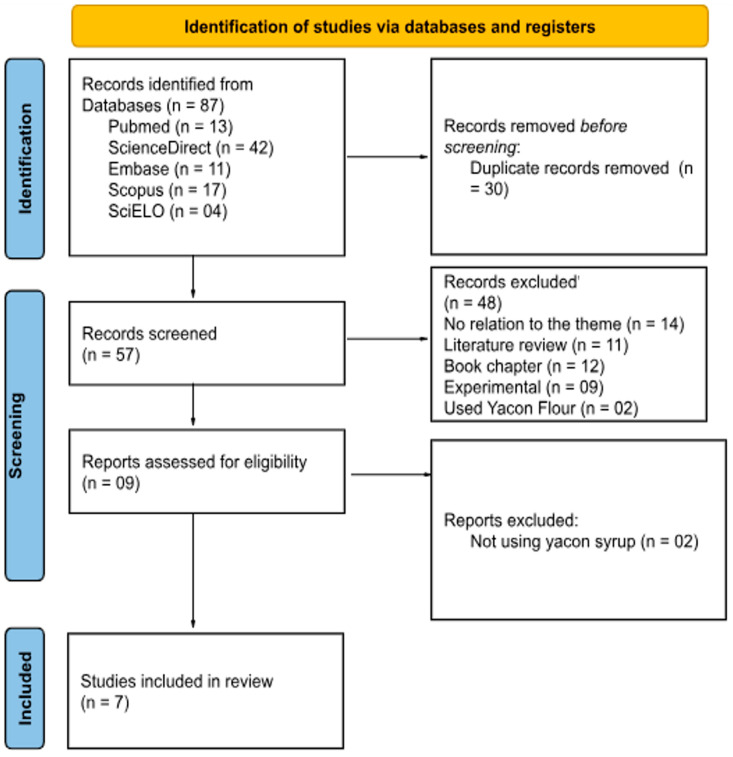
PRISMA diagram showing the screening process.

The initial search across the databases retrieved 87 articles. After removing 30 duplicates, 57 articles remained for title and abstract screening. Then, the inclusion criteria, as mentioned above, were applied and 48 articles were excluded—primarily because they had investigated the sensory acceptability of yacon, focused on osmotic dehydration or production costs, or lacked relevant clinical data. Nine articles were selected for full-text review, and ultimately, seven studies were included in this systematic review. The complete study selection process is summarized in Figure 1. We did not impose any geographical restrictions during the selection process; studies from all regions were considered as long as they met the inclusion criteria. However, no eligible articles were identified outside South America and Europe. Consequently, the final set of included studies is limited to those regions.

### 3.2. Characteristics of Studies and Data Extraction

We summarized important study characteristics to better understand the profile and quality of the included studies in Table 1. Studies were conducted in South America (Brazil, Argentina, and Peru) and Europe (Switzerland). The majority of the studies were randomized, double-blind, placebo-controlled clinical trials. The number of subjects varied between 16 and 55, totaling 161 subjects. The populations studied consist of healthy, overweight, or obese adults, with ages ranging from 18 to 57 years.

The most common dosage in the included studies was 40 g of yacon syrup per day, with FOS concentrations ranging between 6.4 and 14 g. Three clinical trials investigated the acute effect of yacon syrup using a single dose of 40 g (14 g of FOSs), with blood sample collections at intervals of up to 180 min after consumption [29,30,31]. Two other clinical trials evaluated the effects of dietary supplementation with 40 g of yacon syrup (8.74 g of FOSs) per day over two weeks [32,33]. Another study used 20 g of yacon syrup (6.4 g of FOSs) per day for two weeks as well [22]. Lastly, one study evaluated a 120-day intervention, analyzing the daily intake of yacon syrup, with doses of 0.14–0.29 g of FOSs per kg of body weight (b.w.) (approximately 10–20 g FOSs/70 kg b.w.) [21]. These variations in supplementation times and dosages allowed the evaluation of both the immediate effects and long-term impacts of yacon syrup on metabolic parameters, such as glucose and insulin levels, lipid profiles, intestinal microbiota, satiety, colonic transit time, and its adverse effects.

**Table 1:** Characteristics of the included studies.

Study	Country	Population	Study Design	Intervention	Comparison	Duration	Parameters Measured
Sales, 2023 [30]	Brazil	40 women (20 with normal weight and 20 with grade I obesity).	Randomized, crossover, double-blind clinical trial	40 g of yacon syrup (14 g FOSs)	40 g of placebo	2 days, with a 1-week washout period between interventions	BMI, WC, blood glucose, insulin, HOMA-IR, triglycerides, GLP-1, ghrelin, total cholesterol, HDL-C and LDL-C
Adriano, 2020 [29]	Brazil	40 women (20 with normal weight and 20 with grade I obesity).	Randomized, crossover, double-blind clinical trial	40 g of yacon syrup (14 g FOSs)	40 g of placebo	2 days, with a 1-week washout period between interventions	Postprandial ghrelin and GLP-1 concentration and subjective postprandial appetite sensation
Adriano, 2019 [31]	Brazil	40 women (20 with normal weight and 20 with grade I obesity).	Randomized, crossover, double-blind clinical trial	40 g of yacon syrup (14 g FOSs)	40 g of placebo	2 days, with a 1-week washout period between interventions	Postprandial glucose, insulin and triglycerides [24].
Gomes da Silva, 2017 [32]	Brazil	20 healthy adults (10 men, 10 women)	Randomized, parallel, double-blind, placebo-controlled trial	40 g of yacon syrup (8.74 g FOSs)	40 g of corn syrup	2 weeks, and 1 day	Satiety, hunger, fullness, and prospective food consumption using Visual Analogue Scales across multiple time points (0, 30, 60, 90, 120, 150, and 180 min) after breakfast.
Dionísio, 2020 [33]	Brazil	30 healthy subjects (15 in the yacon group and 15 in the placebo group)	Randomized, double-blind, placebo-controlled pilot trial	40 g of yacon syrup (8.74 g FOSs)	40 g of corn syrup	2 weeks	Body weight, WC, BMI, WHR, glucose, insulin, HDL-C, LDL-C, Apo B, triglycerides, LPS, and hsCRP.
Genta, 2009 [21]	Argentina	55 obese women (35 completed the study) aged 31–49 years with mild dyslipidemia and constipation	Randomized, double-blind, placebo-controlled trial	Yacon syrup with two dosage levels: 0.29 g FOSs/kg body weight/day, or 0.14 g FOSs/kg body weight/day.	Placebo syrup	120 days	Body weight, BMI, WC, defecation frequency, serum glucose, serum insulin, HOMA-IR, total cholesterol, LDL-C, HDL-C, and triglycerides.
Geyer, 2008 [22]	Switzerland	16 healthy volunteers (8 males, 8 females) aged 18–57 years	Randomized, double-blind, placebo-controlled, crossover study	20 g of yacon syrup (6.4 g FOSs)	20 g of molasses	2 weeks with a 2-week washout period.	Colonic transit time, stool frequency, stool consistency, and side effects.

Apo B (apolipoprotein B), BMI (body mass index), FOSs (fructooligosaccharides), GLP-1 (glucagon-like peptide 1), HDL-C (HDL cholesterol), HOMA-IR (homeostatic model assessment for insulin resistance), hsCRP (high-sensitivity C-reactive protein), LDL-C (LDL cholesterol), LPS (lipopolysaccharides), WC (waist circumference), WHR (waist–hip ratio).

### 3.3. Study Quality Assessment

**Figure 2:**
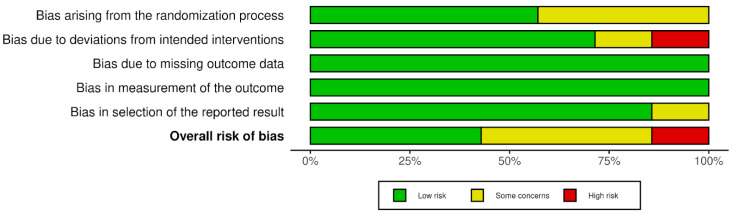
Risk-of-bias graph: assessment regarding each risk-of-bias item across all included studies.

**Figure 3:**
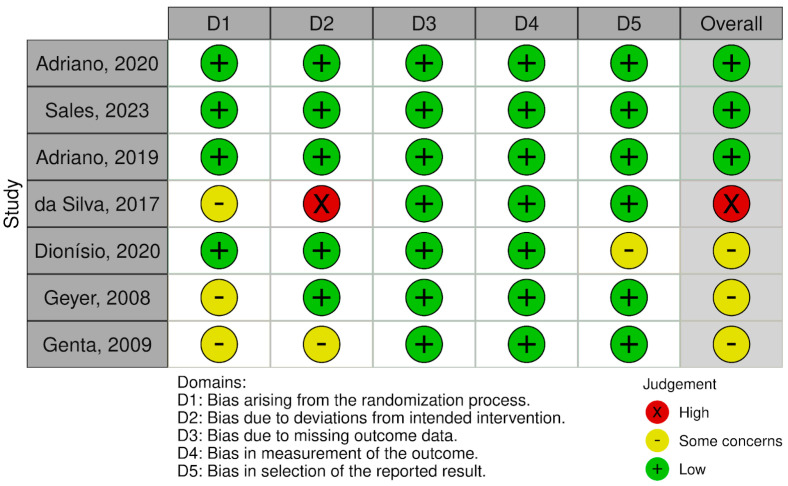
Risk-of-bias summary: assessment regarding each risk-of-bias item for each included study [21,22,29,30,31,32,33].

The quality of the included studies was assessed in terms of risk of bias across multiple domains. Figure 2 and Figure 3 show that the studies generally demonstrated a low risk of “bias arising due to missing outcome data” and “bias in measurement of the outcome”. However, some concerns were noted in “bias due to deviations from intended interventions” and “bias arising from the randomization process”. Three of the studies were assessed as low risk of bias in all domains [29,30,31]. In contrast, one study was classified as high risk in the domain of “deviations from intended interventions” because, although it was described as double-blind, the authors did not clarify how blinding was ensured or whether personnel were truly blinded. Despite no major deviations being reported and an appropriate analysis approach, the lack of transparency on blinding procedures prompted a high-risk assessment in that domain [32]. Overall, these findings still suggest robust design and reporting in most areas across the included studies.

### 3.4. Effects of Yacon Syrup

Table 2 summarizes the main measurable outcomes of each of the included studies.

#### 3.4.1. Impact of Yacon Syrup on Weight Loss and Body Composition

The daily supplementation with yacon syrup (0.14 g of FOSs/kg of body weight) led to a significant reduction in body weight (91.2 ± 8.4 kg to 76.2 ± 6.1 kg, *p* < 0.05), BMI (34 ± 2 kg/m^2^ to 28 ± 3 kg/m^2^, *p* < 0.05), and waist circumference in obese women (105.1 ± 5.0 cm to 95.2 ± 4.8 cm, *p* < 0.05). Over 120 days, participants lost an average of 15 kg, reduced their waist circumference by approximately 10 cm, and experienced a decrease in BMI from 34 to 28 kg/m^2^. These changes were not observed in the placebo group. The study suggests that FOSs in yacon syrup may have played a key role in promoting weight loss, possibly through mechanisms related to increased satiety and the modulation of gastrointestinal peptides, such as GLP-1 [21].

#### 3.4.2. Effects of Yacon Syrup on Satiety and Appetite Control

The effects of yacon syrup on satiety and appetite control were investigated, with results varying depending on the time of consumption. A single dose of 40 g of yacon syrup (containing 14 g of FOSs) in women did not change the postprandial levels of hormones related to appetite: ghrelin (*p* = 0.211 for normal-weight women and *p* = 0.064 for obese women) and GLP-1 (*p* = 0.706 for normal-weight women and *p* = 0.775 for obese women), nor did it change subjective feelings of hunger, satiety, fullness or desire to eat compared with placebo [29]. On the other hand, the daily consumption of 40 g of yacon syrup (with 8.74 g of FOSs) for two weeks increased satiety, especially after 3 h after yacon syrup consumption in a breakfast (*p* < 0.05). After two weeks of consuming yacon syrup daily, healthy women also experienced a greater feeling of fullness (*p* = 0.01). This same study also evaluated the ingestion of a single dose (acute effect) of the syrup, but yacon syrup was not able to change hunger, satiety, or fullness compared with placebo (*p* > 0.05) [32].

#### 3.4.3. Impact of Yacon Syrup on Glycemia and Insulin Resistance

Yacon syrup has shown varying effects on glycemia and insulin resistance, depending on the dosage. For example, the single dose of 40 g of yacon syrup (containing 14 g of FOSs) reduced postprandial glycemia, especially after 30 min of its consumption in healthy women. Also, the acute consumption of yacon syrup decreased the insulin levels at 15, 30, and 45 min after ingestion (*p* < 0.05) [30,31]. Moreover, the daily consumption of yacon syrup (with 0.14 g of FOSs/kg of body weight/day), for a longer period of 120 days, led to a significant reduction in fasting insulin levels (12.6 ± 1.7 to 7.3 ± 2.4 mUI/mL, *p* < 0.05) and HOMA index (6.30 ± 1.10 to 2.07 ± 0.91, *p* < 0.05), suggesting an improvement in insulin resistance in obese women [21]. However, a study evaluating the daily consumption of 40 g of yacon syrup (containing 8.74 g of FOSs) for two weeks in healthy adults did not identify significant differences in fasting glucose levels compared with the placebo group (*p* > 0.05) [33].

The yacon syrup’s effects on glycemia and insulin levels are also dependent on the gut microbiota profile. For example, greater abundance of the phylum *Actinobacteria* and the order *Bifidobacteriales* were positively associated with a better response to yacon syrup in reducing insulin levels [30].

#### 3.4.4. Influence of Yacon Syrup on Lipid Profile

The effects of yacon syrup on the lipid profile were analyzed in three studies included in this review, with results varying according to the duration of the interventions. The daily consumption of 40 g of yacon syrup (containing 8.74 g of FOSs) for 2 weeks did not change total cholesterol, LDL, HDL, or triglycerides levels compared with the placebo group in healthy subjects [33]. Also, a single dose of 40 g of yacon syrup (containing 14 g of FOSs) did not change triglyceride levels in women [31]. However, yacon syrup supplementation (0.14 g of FOSs/kg of body weight/day) for 120 days significantly reduced LDL-cholesterol levels in obese women, suggesting that yacon syrup’s effects on reducing LDL are more evident when given over a longer period [21].

#### 3.4.5. Effects of Yacon Syrup on Gastrointestinal Function

Some studies have investigated the effects of yacon syrup on the intestine, showing promising results. The consumption of 20 g/day of yacon syrup (6.4 g/day of FOSs) for two weeks significantly reduced colonic transit time from 59.7 to 38.4 h in healthy volunteers (*p* < 0.001). There was an increase in the frequency of bowel movements, with a slight trend towards softer stools due to yacon syrup [22]. The long-term study (120 days) also demonstrated that daily consumption of yacon syrup (0.14 g of FOSs/kg of body weight/day) significantly increased the frequency of bowel movements in obese women with a history of constipation (0.28 ± 0.08 to 0.99 ± 0.05 times/day; *p* < 0.05) [21].

However, the high intake of yacon syrup may cause uncomfortable gastrointestinal effects. In total, 60% of subjects who consumed a standard meal plus yacon syrup reported a greater incidence of flatulence compared with 10% of subjects who consumed a standard meal plus placebo. Although the presence of flatulence was more prevalent after consuming yacon syrup, yacon syrup did not provoke abdominal bloating and pain, and there were no changes in stool consistency [31]. Similarly, abdominal swelling was observed, but with no significant difference between yacon syrup and placebo [22]. The uncomfortable gastrointestinal effects due to yacon syrup are not considered serious or harmful to health, and symptoms lessened over time as participants adapted to the yacon syrup consumption [33].

Yacon is well tolerated with no known toxic effects in rats at 340 and 6800 mg FOS/kg body weight/day doses. However, high doses of FOSs can lead to increased flatulence and osmotic pressure, potentially resulting in intestinal discomfort [34]. One study reported that subjects who consumed high concentrations of yacon syrup daily, equivalent to 20 g FOS/70 kg body weight, experienced significant adverse gastrointestinal effects, such as diarrhea, severe abdominal distension, flatulence, and nausea, which led to their exclusion from the study [16]. However, subjects who consumed lower amounts of yacon syrup daily, equivalent to 9.8 g FOSs/70 kg body weight, did not report similar adverse effects. There was no difference in the gastrointestinal effects (flatulence, pain, and abdominal distension *p* > 0.05) and the mean number of days with bloating between the intervention groups. Therefore, the daily consumption of yacon syrup is, overall, well tolerated if FOSs are approximately 10 g or less [21,29].

### 3.5. Production and Marketing of Products with FOSs

**Figure 4:**
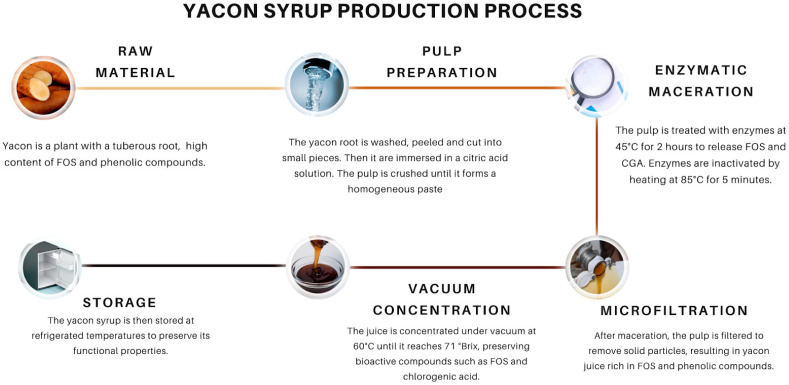
Yacon syrup production process.

Five included studies had similar production of yacon syrup from yacon potato juice, summarized in Figure 4 [29,30,31,32,33]. To prevent the browning of raw yacon potatoes by inactivating polyphenol oxidases, yacon potatoes undergo an acid treatment, causing smaller losses in the content of functional compounds. An enzymatic treatment was also performed with Celluclast^®^ 1.5 L and Pectinex^®^ Ultra SP-L (500 ppm of each enzyme, at 35 °C, 175 rpm, for 2 h) is used to enhance the cellulose and pectin breakdown. Thus, a microfiltration system is used to obtain a clarified juice at temperatures below 60 ± 5 °C. By using vacuum concentration, yacon syrup is created with 71–75% sugar solids (°Br) and 3.7–5.4 pH. Yacon syrup requires a storage temperature of approximately 5 °C. Moreover, the FOS concentration ranges from 22 to 35% of the total mass, 31.9% of glucose, 1.5% of proteins, and 0.07% of lipids. The calorie intake per serving size (40 g) is approximately 67.5 Kcal [33]. All these five studies had the yacon potatoes originated from a local market in Brazil, and the yacon syrup processing was carried out by the Brazilian Agricultural Research Corporation (Embrapa, Fortaleza, Brazil).

Another study was performed in Switzerland, where the yacon syrup processing was carried out at the University Hospital of Basel [22]. In another study using yacon potatoes from Peru, yacon syrup had 41.39% FOSs, 25.65% sugars, 2.16% proteins, and 0.14% lipids [21]. Table 3 presents relevant information on yacon syrup production and analysis mentioned in the respective studies.

According to evidence focused on understanding the nutritional impacts caused by cooking food, potatoes do not experience any loss of phenolic compounds even at temperatures above 100 °C, except in cases of boiling, where those compounds are lost through dilution in the aqueous medium. Placing greater emphasis on potato pulp, an increase in antioxidant activity has also been reported through cooking processes when compared with raw potatoes. This increase has been associated with changes in starch texture, allowing for greater protection of antioxidants, especially when the process involves a potato with its skin intact [35]. Therefore, considering that the production of yacon syrup does not use the boiling method and applies milder temperatures, it is safe to state that the nutritional losses caused by cooking are not significant.

**Table 3:** Summary of yacon syrup production in the included studies.

Study	Processing and Analysis	Source Material
Adriano, 2020 [29] Sales, 2023 [30] Adriano, 2019 [31] Gomes da Silva, 2017 [32] Dionísio, 2020 [33]	Acid and enzymatic treatment, followed by microfiltration, FOS concentration, dissolved sugars (°Br) and pH measurements	Local market in Fortaleza, Ceará, Brazil. Processing carried out at the Brazilian Agricultural Research Corporation (Embrapa) (Fortaleza, Ceará, Brazil)
Genta, 2009 [21]	FOS concentration, dissolved sugars (°Br) and pH measurements, density and proximate composition	International Analytical Laboratories (Lima, Peru)
Geyer, 2008 [22]	Evaporation of liquid extracted from yacon root	Collaboration of a pharmacist responsible for yacon syrup and placebo

As a strategy for FOS supplementation, all studies opted for the direct consumption of yacon syrup associated with common foods. However, Bianchi et al. showed that there are other ways to include yacon syrup in someone’s diet, such as chips, juices, cakes, yogurts, and flour. These different foods expand the use of yacon syrup’s characteristics and palatability [36].

## 4. Discussion

**Figure 5:**
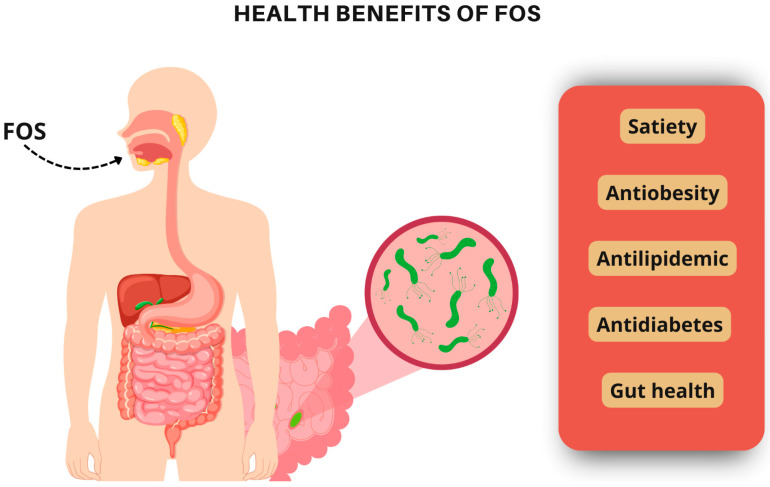
Health benefits of yacon syrup.

In this systematic review, studies reporting the health benefits of yacon syrup consumption were analyzed. Most of the included studies demonstrated a positive relationship between yacon syrup intake and improvements in various metabolic parameters. The observed benefits of yacon syrup are largely attributed to the high concentration of FOS, which appears to play a key role in promoting weight loss and improving several other metabolic parameters (Figure 5). The yacon syrup’s effects occur primarily through increased satiety and the modulation of gastrointestinal peptides, such as GLP-1, which is known to stimulate satiety and reduce food intake. GLP-1 is widely used in the treatment of obesity. Studies show that GLP-1 agonists have demonstrated efficacy in reducing body weight in both patients with diabetes and non-diabetic individuals. These mechanisms, which involve slowing gastric emptying and modulating appetite, may therefore be related to the FOSs present in yacon syrup, reinforcing its impact on weight loss and metabolic control [37].

Furthermore, it was observed that daily consumption of FOSs (8.4 g) for two weeks significantly reduced body fat and increased the levels of leptin, GLP-1, and peptide YY (PYY), hormones involved in appetite control [38]. Similarly, it was shown that daily consumption of FOSs (12 g) for 12 weeks increased GLP-1 levels by 17.42%, reducing body weight, BMI, and hunger [39]. The results of this review reinforce that the effects of yacon syrup on satiety and appetite control depend on the dose and duration of supplementation. For example, a single dose of 40 g of yacon syrup (containing 14 g of FOS) in women did not change the postprandial levels of hormones related to appetite (i.e., ghrelin and GLP-1), nor did it change subjective feelings of hunger, satiety, fullness or desire to eat compared with placebo [29]. On the other hand, the daily consumption of 40 g of yacon syrup (containing 8.74 g of FOSs) for two weeks resulted in greater satiety, especially in women [32]. These findings suggest that chronic and regular FOS supplementation is more effective in appetite control than shorter interventions, likely due to metabolic adaptation that enhances satiety effects. FOSs in yacon syrup can quickly influence gut microbiota, leading to rapid improvements in gut health. This can result in significant short-term benefits, such as enhanced digestion and reduced symptoms of constipation [40]. Although the acute use of yacon syrup does not seem to have a significant impact, longer consumption of yacon syrup shows greater potential for promoting satiety and weight control, particularly in women, reinforcing that regular supplementation is essential to achieving consistent effects [41].

It is known that dietary fibers (e.g., FOSs and inulin) change gut microbiota; however, recent studies reinforce the importance of prior intestinal microbiota profiling to determine the effectiveness of prebiotic interventions. A study conducted with obese patients showed that those with higher levels of *Akkermansia muciniphila* at the start of the intervention showed an improvement in BMI after the use of inulin [38]. Also, the increase in *Faecalibacterium prausnitzii* during the prebiotic intervention was associated with metabolic improvement in some patients [42]. The subject’s gut microbiota profile can significantly influence the metabolic response to the consumption of prebiotics, such as yacon syrup and FOSs. Moreover, recent evidence has shown that habitual dietary fiber intake itself can modulate how individuals respond to inulin-type fructans: those with higher baseline fiber consumption exhibit greater bifidogenic and metabolic changes than low-fiber consumers [43]. Likewise, the dominance of specific bacterial genera (e.g., *Prevotella* versus *Bacteroides*) leads to distinct fermentation patterns and varying SCFA production from the same dietary fiber substrate, reinforcing the need for personalized approaches to prebiotic supplementation [44]. Similarly, a greater abundance of the phylum *Actinobacteria* and the order *Bifidobacteriales* was positively associated with improved insulin response due to yacon syrup intervention, demonstrating that the pre-existing gut microbiota profile impacts the postprandial insulin response following FOS consumption [30]. These factors help to explain the inconsistent effects of yacon syrup on blood glucose and insulin resistance reported in studies. In some cases, the consumption of 8.74 g of FOSs within yacon syrup did not result in significant changes in postprandial glucose levels [28]. On the other hand, acute supplementation with 40 g of yacon syrup (containing 14 g of FOSs) demonstrated a reduction in postprandial glycemia and decreased insulin levels in women [30,31].

The beneficial effects of dietary fibers on glucose metabolism changing the gut microbiota may be related to short-chain fatty acid (SCFA) production [45]. SCFAs play a crucial role in metabolic and intestinal health. For example, butyrate is an important source of energy for colonocytes, while acetate and propionate influence lipid and glucose metabolism by being quickly absorbed into the system [39]. Butyrate also acts directly on brown adipose tissue cells, promoting fatty acid oxidation and reducing insulin resistance. The effects of SCFAs on lipid and glucose metabolism may be mediated by the activation of the AMP-activated protein kinase (AMPK) signaling pathway, which promotes fatty acid oxidation and the phosphorylation of acetyl-CoA carboxylase (ACC), reducing fatty acid synthesis and promoting lipolysis. Moreover, SCFAs modulate glucose metabolism by increasing glucose uptake in muscle cells via the glucose transporter GLUT4 and AMPK. Studies suggest that the activation of AMPK by SCFAs occurs in response to the increase in the AMP/ATP ratio in cells, inducing significant improvements in energy metabolism [46]. Nevertheless, it is yet to be determined whether the increase in SCFAs observed in yacon syrup interventions is unique to this specific food or reflects a general mechanism associated with fibers such as FOSs and inulin. While current evidence suggests that yacon syrup contributes to SCFA production due to its FOS content, further studies are needed to clarify whether this effect is distinct from that of other fiber sources, as well as to evaluate whether its beneficial effects on metabolic parameters are driven by changes in SCFA levels.

The yacon syrup’s ability to favorably promote the growth of gut microbiota results in better gastrointestinal function. These effects are likely due to the FOS content, as FOSs promoted a significant increase in Bifidobacterium while maintaining the constant growth of *Bacteroides*/*Prevotella* in human fecal cultures after 5 h of fermentation [47]. Therefore, these results reinforce the prebiotic effects of yacon syrup and FOSs in improving gastrointestinal function. The prebiotic effects of FOSs help to explain the positive impact of yacon syrup on intestinal health. The consumption of 20 g/day of yacon syrup, equivalent to 6.4 g of FOSs, for two weeks reduced colonic transit time from 59.7 to 38.4 h [22]. The long-term study (120 days) shows that the daily supplementation with yacon syrup (0.14 g of FOSs/kg of body weight/day) significantly increased bowel movement frequency in obese women with a history of constipation. Furthermore, the study demonstrated that regular consumption of yacon syrup promotes the growth of beneficial bacteria in the gut microbiota, leading to improvements in gastrointestinal function [21].

Although there is not strong evidence that yacon syrup has a positive effect on lipid profile, FOSs exert their beneficial effects on lipid profile through several mechanisms that directly impact lipid metabolism. FOS supplementation resulted in a tendency to reduce the levels of total cholesterol, LDL cholesterol, and triglycerides, with a significant increase in HDL cholesterol after 4 weeks [48]. These effects of FOSs and other prebiotics may be due to changes in enzymes responsible for lipogenesis and lipolysis, such as ACC and lipases [49]. The fermentation of FOSs by gut microbiota leading to SCFA production also modulates lipid metabolism. For example, propionate inhibits hepatic cholesterol synthesis and increases cholesterol excretion. Therefore, the ability of FOSs to improve the lipid profile via SCFA may contribute to the prevention of cardiovascular diseases and obesity [47].

Despite the metabolic potential of FOSs, the effects of yacon syrup on the lipid profile appear to vary depending on the duration of interventions and the characteristics of the studied population. For example, a study with 30 healthy individuals consuming 40 g of yacon syrup (containing 8.74 g of FOSs) per day for two weeks [33], and another study with 40 women evaluating the impact of a single dose of 40 g of yacon syrup (containing 14 g of FOSs) [32], found no significant changes in lipid parameters. However, more consistent evidence was observed in longer interventions, where daily consumption of yacon syrup (0.14 g of FOSs/kg of body weight/day) for 120 days led to a reduction in LDL cholesterol in obese women [21]. Similarly, a 30-day intervention with yacon syrup (12 mL/day, providing 5.3 g of FOSs) led to a reduction in total cholesterol and body fat percentage in overweight and obese women, without significant changes in blood glucose levels [50]. These results suggest that the benefits of yacon syrup on the lipid profile, particularly in reducing LDL cholesterol, are more evident in prolonged interventions and in specific populations, such as obese individuals. Obese individuals often have altered gut microbiota and metabolic profiles compared with non-obese individuals. The prebiotic properties of yacon syrup may help restore balance to the gut microbiome, which can improve metabolic health, including lipid profiles, in these populations [50].

Knowing that the planetary health diet encourages increased consumption of plant-based foods that have physiological and biochemical benefits, Yacon syrup is related to this plant-based diet both due to its bioactive compounds that bring health benefits, as well as because its use supports sustainable practices, which is directly related to the planetary health diet [20]. Furthermore, this review highlights the relevance of Yacon syrup and its benefits for human health, highlighting the need for further research to explore and clarify its effects more comprehensively.

Although the findings are promising, some limitations should be acknowledged. First, sample sizes in most studies were relatively small, ranging from 16 to 55 participants [21,22]. The limited number of participants may affect the statistical power and the generalizability of the results. Additionally, most studies were conducted in specific populations, limiting the applicability of the findings to other groups that could benefit from the consumption of yacon syrup, such as men, older adults, and individuals with type 2 diabetes [4]. Another limitation is that other hormones, such as CCK and PYY, were not analyzed, which could have provided further insights into the mechanisms involved. Additionally, SCFAs themselves have been linked to elevated circulating levels of peptide YY (PYY) [51]. We suggest that yacon syrup may be a promising alternative for weight control and metabolic health, primarily due to its prebiotic effects and impact on appetite regulation. Its metabolic benefit appears to be dose-dependent and influenced by gut microbiota, reinforcing the need for personalized nutritional approaches. Future research should explore whether specific gut microbiota profiles can predict metabolic responses to yacon syrup and whether targeted probiotic or dietary strategies can enhance its benefits.

## 5. Conclusions

The studies analyzed in this systematic review demonstrate that yacon syrup has promising effects on improving metabolic parameters, including reducing body weight and waist circumference, as well as improving insulin sensitivity. Yacon syrup also has a positive effect on satiety and improved intestinal health. However, there is still debate over the effective and ideal dose and duration of yacon syrup consumption to maximize its benefits. Also, the response to yacon syrup may be influenced by individual microbiota composition, metabolic status, and the duration of the intervention. Further human clinical trials are needed to confirm and explore other yacon syrup functional properties, such as its potential use as an anti-obesity intervention.

## Figures and Tables

**Table 2:** Main outcomes of included studies.

Study	Main Outcomes
Adriano, 2020 [29]	The ingestion of yacon syrup (40 g with 14 g of FOSs) showed no significant effects on postprandial levels of ghrelin (*p* = 0.211 for normal-weight women and *p* = 0.064 for obese women) and GLP-1 (*p* = 0.706 for normal-weight women and *p* = 0.775 for obese women). There was no significant impact on subjective appetite sensations, including hunger, satiety, and desire to eat (*p* > 0.05) across all time points and groups. No significant gastrointestinal effects (flatulence, abdominal discomfort, distension) were observed [27].
Sales, 2023 [30]	Yacon syrup (40 g containing 14 g of FOSs) reduced postprandial insulin levels compared with placebo at the 30 min mark (*p* < 0.05). Responders had higher abundances of *Actinobacteria* (*p* = 0.021) and *Bifidobacteriales* (*p* = 0.013), indicating that prior gut microbiota composition influences the insulin response to yacon syrup. Six non-responders were identified based on elevated insulin levels, with higher BMI and WC than responders (*p* < 0.001). No significant gastrointestinal effects (flatulence, abdominal discomfort, distension) were observed [28].
Adriano, 2019 [31]	Yacon syrup (40 g containing 14 g of FOSs) reduced postprandial glucose levels at 30 min (*p* < 0.01) and insulin levels at 15, 30, and 45 min (*p* < 0.05) compared with placebo. No significant effect was observed on triglycerides levels (*p* > 0.05). Flatulence is more prevalent in the yacon syrup group, but there is no significant difference for abdominal pain or distension [29].
Gomes da Silva, 2017 [32]	Yacon syrup (8.74 g FOSs/day) over two weeks showed significant increases in satiety and fullness only in women, with *p*-values indicating gender-specific responses. The overall yacon syrup effect for satiety had *p* = 0.059, and satiety significant increased at the 180 min mark (*p* < 0.05). For fullness, there was a significant treatment effect in women (*p* = 0.01). No adverse effects reported.
Dionísio, 2020 [33]	Yacon syrup (40 g/day containing 8.74 g FOSs) over two weeks showed no significant effect on fasting glucose (*p* > 0.05), total cholesterol (*p* > 0.05), HDL-C (*p* > 0.05), LDL-C (*p* > 0.05), triglycerides (*p* > 0.05), insulin levels (*p* > 0.05), or LPS (*p* > 0.05). No changes in BMI, WC, nor WHR were observed (*p* > 0.05). Mild gastrointestinal discomfort was initially observed but resolved over time. Symptoms such as flatulence, bloating, and abdominal rumbling, decreased after the first few days. None of these symptoms were considered serious or harmful.
Genta, 2009 [21]	Yacon syrup (0.14 g FOSs/kg body weight/day) over 120 days in obese pre-menopausal women significantly decreased body weight (91.2 ± 8.4 kg to 76.2 ± 6.1 kg, *p* < 0.05), BMI (34 ± 2 to 28 ± 3, *p* < 0.05), and WC (105.1 ± 5.0 cm to 95.2 ± 4.8 cm, *p* < 0.05). Significant reductions were observed in fasting insulin (12.6 ± 1.7 to 7.3 ± 2.4 mUI/mL, *p* < 0.05) and HOMA-IR (6.30 ± 1.10 to 2.07 ± 0.91, *p* < 0.05), indicating improved insulin sensitivity. Defecation frequency increased from 0.28 ± 0.08 to 0.99 ± 0.05 times/day (*p* < 0.05). No significant changes in fasting glucose or lipid levels, except for LDL-C reduction (*p* < 0.05). Mild gastrointestinal effects such as abdominal distension and flatulence were observed in the higher-dose group (0.29 g FOSs/kg body weight/day), leading to exclusion from the analysis. The group treated with yacon syrup at a level intake of 0.14 g FOSs/kg body weight/day went through the whole experimental period with no difficulties.
Geyer, 2008 [22]	Yacon syrup (20 g/day containing 6.4 g FOSs) over a two-week period significantly decreased colonic transit time from 59.7 ± 4.3 h to 38.4 ± 4.2 h (*p* < 0.001), mainly due to an accelerated transit in the right colon (*p* = 0.013). There was a slight, non-significant increase in stool frequency (1.1 to 1.3 times/day, *p* = 0.17) and softer stool consistency (*p* = 0.48). The syrup was well tolerated with no severe adverse effects. Bloating was noted but was not significantly different between yacon syrup and placebo.

BMI (body mass index), FOSs (fructooligosaccharides), GLP-1 (glucagon-like peptide 1), HDL-C (HDL cholesterol), HOMA-IR (homeostatic model assessment for insulin resistance), LDL-C (LDL cholesterol), LPS (lipopolysaccharides), WC (waist circumference), WHR (waist–hip ratio).

## Supplementary Materials

The following material is available online at https://www.mdpi.com/article/10.3390/nu17050888/s1: Table S1: PRISMA 2020 Checklist, which details the systematic review process followed in this study.

## References

[1] Hong S.S., Lee S.A., Han X.H., Lee M.H., Hwang J.S., Park J.S., Oh K.-W., Han K., Lee M.K., Lee H. (2008). Melampolides from the Leaves of *Smallanthus sonchifolius* and Their Inhibitory Activity of LPS-Induced Nitric Oxide Production. Chem. Pharm. Bull..

[2] Ojansivu I., Ferreira C.L., Salminen S. (2011). Yacon, a New Source of Prebiotic Oligosaccharides with a History of Safe Use. Trends Food Sci. Technol..

[3] Ashraf A., Rasool M.H., Shahzadi M., Irshad Z., Saeed S., Wahab A. (2022). Exploring Anti-Diabetes Potential of Yacon Powder in Elderly Subjects. Int. J. Sci. Res. Publ..

[4] Caetano B.F.R., De Moura N.A., Almeida A.P.S., Dias M.C., Sivieri K., Barbisan L.F. (2016). Yacon (*Smallanthus sonchifolius*) as a Food Supplement: Health-Promoting Benefits of Fructooligosaccharides. Nutrients.

[5] Ricarte D., de Almeida Júlio B.L., Zocateli G.A.F., Barreto R.L.F., Guimarães M., de Souza Ferreira R., Guimarães N.S. (2019). Análise Sensorial de Preparações Com Batata Yacon: Revisão Sistemática. HU Rev..

[6] Duarte M.d.R., Wolf S., de Paula B.G. (2008). *Smallanthus sonchifolius* (Poepp.) H. Rob.(Yacón): Identificação Microscópica de Folha e Caule Para o Controle de Qualidade Farmacognóstico. Rev. Bras. Cienc. Farm..

[7] Valentová K., Cvak L., Muck A., Ulrichova J., Simanek V. (2003). Antioxidant Activity of Extracts from the Leaves of *Smallanthus sonchifolius*. Eur. J. Nutr..

[8] Valentová K., Moncion A., De Waziers I., Ulrichova J. (2004). The Effect of *Smallanthus sonchifolius* Leaf Extracts on Rat Hepatic Metabolism. Cell Biol. Toxicol..

[9] Joung H., Kwon D.-Y., Choi J.-G., Shin D.-Y., Chun S.-S., Yu Y.-B., Shin D.-W. (2010). Antibacterial and Synergistic Effects of *Smallanthus sonchifolius* Leaf Extracts against Methicillin-Resistant *Staphylococcus aureus* under Light Intensity. J. Nat. Med..

[10] de Andrade E.F., de Souza Leone R., Ellendersen L.N., Masson M.L. (2014). Phenolic Profile and Antioxidant Activity of Extracts of Leaves and Flowers of Yacon (*Smallanthus sonchifolius*). Ind. Crops Prod..

[11] Gonzales M.G., Del Rosario R.M., Walag A.M.P. (2023). Proximate Biochemical Composition and Antinutritional Analyses of the Selected Parts of Yacon (*Smallanthus sonchifolius*). Asian J. Biol. Life Sci..

[12] Chessum K., Chen T., Kam R., Yan M. (2022). A Comprehensive Chemical and Nutritional Analysis of New Zealand Yacon Concentrate. Foods.

[13] Rocha M.A., de Oliveira V.P. (2013). Efeitos Da Inulina Sobre o Perfil Glicêmico Em Ratos Induzidos Ao Diabetes Mellitus Tipo 2. Rev. Cient. Faminas.

[14] Costa G., Vasconcelos Q., Abreu G., Albuquerque A., Vilarejo J., Aragão G. (2020). Changes in nutrient absorption in children and adolescents caused by fructans, especially fructooligosaccharides and inulin. Arch. Pediatr..

[15] Alles M.S., de Roos N.M., Bakx J.C., van de Lisdonk E., Zock P.L., Hautvast J.G. (1999). Consumption of fructooligosaccharides does not favorably affect blood glucose and serum lipid concentrations in patients with type 2 diabetes. Am. J. Clin. Nutr..

[16] Campos-Perez W., Martinez-Lopez E. (2021). Effects of short chain fatty acids on metabolic and inflammatory processes in human health. BBA-Mol. Cell Biol. Lipids.

[17] Gong S., Yang J., Zhang J., Wu X., Jiang S., Zhang Y., Gong G., Wu N., Sun J., Wu Z. (2023). Yacon Root Extract Improves Lipid Metabolism in Hyperlipidemic Rats by Inhibiting HMGCR Expression and Activating the PPAR α/CYP7A1/CPT-1 Pathway. Nan Fang Yi Ke Da Xue Xue Bao.

[18] Honoré S.M., Grande M.V., Gomez Rojas J., Sánchez S.S. (2018). *Smallanthus sonchifolius* (Yacon) Flour Improves Visceral Adiposity and Metabolic Parameters in High-Fat-Diet-Fed Rats. J. Obes..

[19] Rahmaisyah D., Wasityastuti W., Astarini F.D., Widasari D.I. (2022). Protective Effects of Yacon Syrup Powder on Colonic Interleukin-23 and Leukocyte Infiltration Profile in TNBS-Induced Colitis Mouse Model. Med. J. Nutr. Metab..

[20] Yan M.R., Welch R., Rush E.C., Xiang X., Wang X. (2019). A Sustainable Wholesome Foodstuff; Health Effects and Potential Dietotherapy Applications of Yacon. Nutrients.

[21] Genta S., Cabrera W., Habib N., Pons J., Carillo I.M., Grau A., Sánchez S. (2009). Yacon Syrup: Beneficial Effects on Obesity and Insulin Resistance in Humans. Clin. Nutr..

[22] Geyer M., Manrique I., Degen L., Beglinger C. (2008). Effect of Yacon (*Smallanthus sonchifolius*) on Colonic Transit Time in Healthy Volunteers. Digestion.

[23] Koumpouli D., Koumpouli V., Koutelidakis A.E. (2024). Functional Foods, Gut Microbiome and Association with Obesity and Metabolic Syndrome: A Literature Review. Appl. Sci..

[24] Silva I.F., Bragante W.R., Junior R.C.M., Laurindo L.F., Guiguer E.L., Araújo A.C., Fiorini A.M.R., Nicolau C.C.T., Oshiiwa M., Lima E.P. (2024). Effects of *Smallanthus sonchifolius* Flour on Metabolic Parameters: A Systematic Review. Pharmaceuticals.

[25] Page M.J., McKenzie J.E., Bossuyt P.M., Boutron I., Hoffmann T.C., Mulrow C.D., Shamseer L., Tetzlaff J.M., Akl E.A., Brennan S.E. (2021). The PRISMA 2020 statement: An updated guideline for reporting systematic reviews. BMJ.

[26] Moher D., Liberati A., Tetzlaff J., Altman D.G., PRISMA Group (2009). Preferred reporting items for systematic reviews and meta-analyses: The PRISMA statement. PLoS Med..

[27] Ouzzani M., Hammady H., Fedorwicz Z., Elmagarmid A. (2016). Rayyan—A web and mobile app for systematic reviews. Syst. Rev..

[28] Cumpston M., Li T., Page M.J., Chandler J., Welch V.A., Higgins J.P., Thomas J. (2019). Updated guidance for trusted systematic reviews: A new edition of the Cochrane Handbook for Systematic Reviews of Interventions. Cochrane Database Syst. Rev..

[29] Adriano L.S., Dionísio A.P., Pinto de Abreu F.A., Wurlitzer N.J., Cordeiro de Melo B.R., Ferreira Carioca A.A., de Carvalho Sampaio H.A. (2020). Acute postprandial effect of yacon syrup ingestion on appetite: A double-blind randomized crossover clinical trial. Food Res. Int..

[30] da Silva Sales S., Dionísio A.P., Adriano L.S., de Melo B.R.C., de Abreu F.A.P., de Carvalho Sampaio H.A., Carioca A.A.F. (2023). Previous gut microbiota has an effect on postprandial insulin response after intervention with yacon syrup as a source of fructooligosaccharides: A randomized, crossover, double-blind clinical trial. Nutrition.

[31] Adriano L.S., Dionísio A.P., Abreu F.A.P., Carioca A.A.F., Zocolo G.J., Wurlitzer N.J., Pinto C.O., de Oliveira A.C., Sampaio H.A.C. (2019). Yacon syrup reduces postprandial glycemic response to breakfast: A randomized, crossover, double-blind clinical trial. Food Res. Int..

[32] Gomes da Silva M.F., Dionísio A.P., Ferreira Carioca A.A., Silveira Adriano L., Pinto C.O., Pinto de Abreu F.A., Wurlitzer N.J., Araújo I.M., Dos Santos Garruti D., Ferreira Pontes D. (2017). Yacon syrup: Food applications and impact on satiety in healthy volunteers. Food Res. Int..

[33] Dionísio A.P., Silva M.d.F.G.d., Carioca A.A.F., Adriano L.S., de Abreu F.A.P., Wurlitzer N.J., Pinto C.d.O., Pontes D.F. (2020). Effect of yacon syrup on blood lipid, glucose and metabolic endotoxemia in healthy subjects: A randomized, double-blind, placebo-controlled pilot trial. Food Sci. Technol..

[34] Genta S.B., Cabrera W.M., Grau A., Sánchez S.S. (2005). Subchronic 4-month oral toxicity study of dried *Smallanthus sonchifolius* (yacon) roots as a diet supplement in rats. Food Chem. Toxicol..

[35] Lopes M.C., Silva G.C., Tagliapietra B.L. (2024). Impact of cooking methods on the availability of nutrients in foods of plant origin. Discip. Sci. Saúde.

[36] Bianchi A.P., Felipe M.R., Malaquias P.S., Centurion E.B.E. (2021). Efeito da batata yacon (*Smallanthus sonchifolia*) sobre os parâmetros glicêmicos de idosos institucionalizados. Rev. Assoc. Bras. Nutr. RASBRAN.

[37] Popoviciu M.-S., Păduraru L., Yahya G., Metwally K., Cavalu S. (2023). Emerging Role of GLP-1 Agonists in Obesity: A Comprehensive Review of Randomised Controlled Trials. Int. J. Mol. Sci..

[38] Arcos Castellanos L., López Plaza B., Morato Martínez M., Valero Pérez M., Palma Milla S., Gómez Candela C. (2022). El consumo regular de un caldo funcional enriquecido con fosfofructooligosacáridos aumenta los niveles de las hormonas relacionadas con la saciedad en las personas sanas: Ensayo clínico aleatorizado y controlado. Nutr. Hosp..

[39] Sheth M., Gupta N. (2014). Metabolic Effect of FOS (Fructooligosaccharide) in Terms of Gut Incretin (GLP-1), Gut Microflora and Weight Reduction in Obese Adults. Int. J. Appl. Biol. Pharm. Technol..

[40] Torres-Valenzuela L.S., Villamizar R., Ángel-Rendón S. (2014). Stabilization of a functional refreshment from mango nectar and yacon (*Smallanthus sonchifolius*) through spray drying encapsulation. Funct. Foods Health Dis..

[41] Assudani A.D. (2019). Acceptability Trials of Fructooligosaccharide (FOS) Added Popular Indian Recipes and Impact Evaluation of FOS Intervention in Modulating Gut Microflora, Gut Satietogenic Hormones and Anthropometric Indices of Young Obese Bank Employees of Urban Vadodara: A Fat–Fit Study.

[42] Rodriguez J., Hiel S., Neyrinck A.M., Le Roy T., Pötgens S.A., Leyrolle Q., Pachikian B.D., Gianfrancesco M.A., Cani P.D., Paquot N. (2020). Discovery of the Gut Microbial Signature Driving the Efficacy of Prebiotic Intervention in Obese Patients. Gut.

[43] Healey G., Murphy R., Butts C., Brough L., Whelan K., Coad J. (2018). Habitual dietary fibre intake influences gut microbiota response to an inulin-type fructan prebiotic: A randomised, double-blind, placebo-controlled, cross-over, human intervention study. Br. J. Nutr..

[44] Chen T., Long W., Zhang C., Liu S., Zhao L., Hamaker B.R. (2017). Fiber-utilizing capacity varies in Prevotella- versus Bacteroides-dominated gut microbiota. Sci. Rep..

[45] Poeker S.A., Geirnaert A., Berchtold L., Greppi A., Krych L., Steinert R.E., de Wouters T., Lacroix C. (2018). Understanding the Prebiotic Potential of Different Dietary Fibers Using an In Vitro Continuous Adult Fermentation Model (PolyFermS). Sci. Rep..

[46] Blaak E.E., Canfora E.E., Theis S., Frost G., Groen A.K., Mithieux G., Nauta A., Scott K., Stahl B., van Harsselaar J. (2020). Short Chain Fatty Acids in Human Gut and Metabolic Health. Benef. Microbes.

[47] Hajar-Azhari S., Abd Rahim M.H., Sarbini S.R., Muhialdin B.J., Olusegun L., Saari N. (2021). Enzymatically Synthesised Fructooligosaccharides from Sugarcane Syrup Modulate the Composition and Short-Chain Fatty Acid Production of the Human Intestinal Microbiota. Food Res. Int..

[48] Kim E.J., Kim M.Y., Kim J.S., Cho K.D., Han C.K., Lee B.H. (2011). Effects of Fructooligosaccharides Intake on Body Weight, Lipid Profiles, and Calcium Status among Korean College Students. FASEB J..

[49] dos Reis S.A., da Conceição L.L., Rosa D.D., dos Santos Dias M.M., Peluzio M.C.G. (2015). Mechanisms Used by Inulin-Type Fructans to Improve the Lipid Profile. Nutr. Hosp..

[50] Cabral M.M., Carioca A.A.F., Sousa G.M.A., Freitas P.A., Tahim J.C., Garces T.S., Moreira T.M.M., Magalhães S.C., Souza A.N.C., Oliveira A.C. (2024). Yacon Syrup Supplementation Improves Cholesterol and Body Composition in Overweight and Obese Women: A Double-Blind Randomized Clinical Trial. Nutrivisa.

[51] Byrne C.S., Chambers E.S., Morrison D.J., Frost G. (2015). The role of short chain fatty acids in appetite regulation and energy homeostasis. Int. J. Obes..

